# The standardized *Withania somnifera* Dunal root extract alters basal and morphine-induced opioid receptor gene expression changes in neuroblastoma cells

**DOI:** 10.1186/s12906-017-2065-9

**Published:** 2018-01-10

**Authors:** Francesca Felicia Caputi, Elio Acquas, Sanjay Kasture, Stefania Ruiu, Sanzio Candeletti, Patrizia Romualdi

**Affiliations:** 1 Department of Pharmacy and Biotechnology, Alma Mater Studiorum - University of Bologna, Via Irnerio 48, 40126 Bologna, Italy; 2 Department of Life & Environmental Sciences, University of Cagliari, Via Ospedale, 72, 09124 Cagliari, Italy; 3 Pinnacle Biomedical Research Institute, Bhopal, India; 4 National Research Council (C.N.R.) - Institute of Translational Pharmacology, U.O.S. of Cagliari, Science and Technology Park of Sardinia Polaris, Pula, Italy

**Keywords:** *Withania somnifera*, MOP receptors, NOP receptors, SH-SY5Y cells

## Abstract

**Background:**

Behavioral studies demonstrated that the administration of *Withania somnifera* Dunal roots extract (WSE), prolongs morphine-elicited analgesia and reduces the development of tolerance to the morphine’s analgesic effect; however, little is known about the underpinning molecular mechanism(s). In order to shed light on this issue in the present paper we explored whether WSE promotes alterations of μ (MOP) and nociceptin (NOP) opioid receptors gene expression in neuroblastoma SH-SY5Y cells.

**Methods:**

A range of WSE concentrations was preliminarily tested to evaluate their effects on cell viability. Subsequently, the effects of 5 h exposure to WSE (0.25, 0.50 and 1.00 mg/ml), applied alone and in combination with morphine or naloxone, on MOP and NOP mRNA levels were investigated.

**Results:**

Data analysis revealed that morphine decreased MOP and NOP receptor gene expression, whereas naloxone elicited their up-regulation. In addition, pre-treatment with naloxone prevented the morphine-elicited gene expression alterations. Interestingly, WSE was able to: a) alter MOP but not NOP gene expression; b) counteract, at its highest concentration, morphine-induced MOP down-regulation, and c) hamper naloxone-induced MOP and NOP up-regulation.

**Conclusion:**

Present in-vitro data disclose novel evidence about the ability of WSE to influence MOP and NOP opioid receptors gene expression in SH-SY5Y cells. Moreover, our findings suggest that the in-vivo modulation of morphine-mediated analgesia by WSE could be related to the hindering of morphine-elicited opioid receptors down-regulation here observed following WSE pre-treatment at its highest concentration.

## Background

The use of herbal preparations in folk medicine has traditionally ancient roots and since often it is not fully scientifically validated an increasing effort is currently required to bridge this gap. Accordingly, in the last decades a high number of herbal preparations with specific indications, including anti-inflammatory [1], anti-microbial [2], anti-spastic [3], anti-arrhythmic [4] and anti-depressant [5] activities, just to name a few, has been examined and their chemical composition and mechanism(s) of action have been investigated in great detail. Interestingly, instances of different therapeutic properties coexisting in the same plant have been reported, and among the most promising herbs endowed with this feature *Withania somnifera* Dunal has shown an exponential growth in terms of scientific interest [6–8]. The number of records in PubMed for the key word “*Withania somnifera*” has considerably increased from 43 between 1990 and 2000 [9, 10], to 275 in the period relative to 2000 – 2010 [11, 12]. In addition, the 566 studies collected in the period relative to 2010 – 2017 [13, 14] (up to the time of preparation of the manuscript) represent more than half of the total 915 citations found with this key word search.

*Withania somnifera,* or Ashwagandha, is an evergreen shrub native of the Indian subcontinent which spontaneously grows also in the Mediterranean basin [15]. Its increasing attractiveness is mostly due to the anti-inflammatory [1, 8] and anti-cancer [1, 6] properties, but also to a number of central effects related to stress [8], anxiety [16] and neurodegenerative disorders [8, 17]. In this frame, it is worth noting that Withanolides and Withaferin A, abundantly present in *Withania somnifera* roots, have been reported to interact with cholinergic mechanisms [18] and also with Nuclear factor-κB [19–21].

Previous studies from our and other laboratories have shown that the standardized methanolic extract of *Withania somnifera* roots (WSE) prevents **i)** the dendritic spine density reduction in the shell of nucleus accumbens of rats undergoing morphine withdrawal [22], **ii)** the acquisition and expression of morphine-elicited conditioned place preference [23] and **iii)** the development of tolerance to the analgesic effects of morphine [24]. Moreover, we recently reported that WSE, although lacking of analgesic properties on its own, prolongs the anti-nociceptive effect of morphine and counteracts the paradoxical morphine-induced hyperalgesia in CD-1 mice [25], suggesting that WSE could represent a valuable adjuvant in opioid-sparing pain therapies. In addition, we assessed the binding affinities for a number of receptors [23, 25] and we found that WSE shows moderate affinities for GABA_A_ and GABA_B_ (13 and 130 μg/ml, respectively) as well as for opioid [μ (MOP): 385 μg/ml; δ: 166 μg/ml; κ: 775 μg/ml] receptors.

Interestingly, the absence of analgesic activity of WSE on its own, together with its low affinity for MOP receptors, suggest that the mentioned WSE/morphine interplay [22–25] might involve molecular mechanisms different from a direct receptor interaction. In this regard, gene expression alteration has been suggested as a likely mechanism for inducing long-term neuroadaptations responsible for tolerance development [26]. Furthermore, the opioid receptor gene expression regulation is differently affected by diverse opioid ligands such as morphine, fentanyl and tapentadol [27–29] which in turn recruit different G protein-coupled receptor kinase isoforms, as well as exhibit diverse tolerance profiles [29–31].

Based on these premises, the present study was designed to verify whether the behavioral outcomes deriving from the interaction between WSE and morphine [24, 25] might be related to changes in gene expression of MOP and/or nociceptin- opioid (NOP) receptor genes, both deeply involved in the regulation of morphine analgesic properties. Notably, a critical role has been attributed to NOP receptor in several mechanisms such as desensitization, down-regulation [32–34] and intracellular signal transduction pathways [35, 36], involved in the analgesic responses to endogenous or exogenous opioid ligands [37].

Hence, using the validated in-vitro model represented by the SH-SY5Y neuroblastoma cells expressing MOP and NOP receptors [28, 38], we aimed at evaluating whether cell exposure to different WSE concentrations affects MOP and NOP gene expression. Furthermore, since MOP and NOP mRNA levels can be altered by both morphine and naloxone [27], the effect of concomitant exposure to WSE plus morphine or naloxone was also examined.

## Methods

### Cell culture

Human SH-SY5Y neuroblastoma cells purchased from ICLC-IST (Genoa, Italy), were cultured in Dulbecco’s modified Eagle medium (DMEM), supplemented with 10% (*v*/v) fetal bovine serum (FBS), 100 units/ml penicillin, 100 μg/ml streptomycin and 2 mM glutamine. Cells were incubated at 37 °C in a humidified atmosphere containing 5% CO_2_ and then were allowed to reach 80% confluence before starting treatments. For each analysis new cell sets were plated. All reagents for cell culture were purchased from Lonza (Milan, Italy).

### Drugs and cell culture treatments

Morphine hydrochloride was supplied by Carlo Erba (Milan, Italy); naloxone hydrochloride was supplied by Research Biochemicals INC. (Cambridge, UK). WSE, previously authenticated (NISCAIR, New Delhi, India), was kindly provided by Natural Remedies Pvt. Ltd., Bangalore, India. All substances were dissolved in DMEM and the SH-SY5Y cells were exposed to different treatment schedules. Firstly, a range of WSE concentrations (0.10–0.25-0.50-0.75 mg/ml and 1.00 mg/ml) was tested in a cell viability assay to rule out toxic effects that might affect the interpretation of the findings.

Secondly, the effects elicited by 10 μM morphine, 100 μM naloxone, or by their association on MOP and NOP gene expression were evaluated (treatment A - see Table 1).

**Table 1:** Cell culture treatments

Treatment	Drug	Concentration	Time
A	Morphine	10 μM	5 h
	Naloxone	100 μM	
	Naloxone (30 min before)	100 μM	
	+		
	Morphine	10 μM	
B	WSE	0.25 mg/ml	
		0.50 mg/ml	
		1.00 mg/ml	
C	WSE (30 min before)	0.25 mg/ml	
		0.50 mg/ml	
		1.00 mg/ml	
	+		
	Morphine	10 μM	
D	WSE	0.25 mg/ml	
		0.50 mg/ml	
		1.00 mg/ml	
	+		
	Naloxone	100 μM	

WSE: *Withania somnifera* root extract

Thirdly, the alterations of MOP and NOP mRNA levels induced by 0.25 mg/ml, 0.50 mg/ml and 1.00 mg/ml WSE were assessed (treatment B - see Table 1). Fourthly, the effects elicited by WSE together with morphine (treatment C - see Table 1) or naloxone (treatment D - see Table 1) were ascertained.

### WSE extraction and high-performance liquid chromatography (HPLC) analysis

Shade dried roots of *Withania somnifera* Dunal (1 Kg) were extracted in methyl alcohol (5 L) using Soxhlet’s extractor apparatus (Borosil Glass Works Ltd., Ahmedabad, India). The extraction was prolonged until the liquid in siphon tube of Soxhlet’s extractor did not show any spot of extract on the thin layer chromatography plate, developed using methanol as a solvent system.

The extract was dried under vacuum below 40 °C yielding 20.1% of the extract.

Then the extract was dissolved in methyl alcohol (10 mg/ml) and subsequently characterized by an HPLC-fingerprint analysis, as certified by Natural Remedies Pvt. Ltd., with identification of the main withanolides present in the extract. This analysis, with the necessary description of the technique, has been previously reported [22]. Compounds isolated from the extract and characterized as given in the literature [39], were used as reference standard for HPLC analysis: 20 μl of the mixed standard solution (≅ 100 μg/ml of each Withanolide in methyl alcohol) and sample solution (10 mg/ml in methanol). A HPLC system (Shimadzu, LC 2010 A, Japan) equipped with UV detector, auto-injector, and column oven with class VP software was used.

The stationary phase was an octadecylsilane column (Phenomenex-Luna, C18, 5 μm, 250 mm × 4.6 mm). The mobile phase was a mixture of phosphate buffer [prepared by dissolving 0.136 g of KH_2_PO_4_ in 900 ml of HPLC grade water and by adding 10% aqueous H_3_PO_4_ (pH adjusted to 2.8 ± 0.05) and making the volume of 1000 ml with water, Solvent A] and acetonitrile (Solvent B) (HPLC grade, Qualigens). The flow rate of mobile phase was maintained at 1.5 ml/min throughout the analysis and the detector wave length was kept at 227 nm. Acetonitrile and phosphate buffer were mixed and the solvent B concentration was increased as linear gradient in the first 18 min from 5 to 45% and from the 18th to 25th min from 45 to 80%.

### Cell viability assay

Cell viability was measured using the MTT [3-(4,5-dimethylthiazol-2-yl)-2,5-diphenyltetrazolium bromide] assay [40]. All reagents were purchased from Sigma-Aldrich (Milan, Italy) unless otherwise indicated. Briefly, cells were plated on 24-well plates at a density of 3 × 10^4^ cells/well, and were grown to reach 80% confluence. Cells were treated with a solution of WSE in DMEM (0.10 mg/ml, 0.25 mg/ml, 0.50 mg/ml, 0.75 mg/ml and 1.00 mg/ml), or vehicle (DMEM). After 5 h, 24 h or 48 h, the culture medium was removed and replaced with fresh medium containing the MTT solution (0.5 mg/mL) and cells were incubated in the dark at 37 °C for 3 h. After supernatant removal, a dimethyl sulfoxide-ethanol (4:1) mixture was added to each well to dissolve formazan crystals. The optical densities (OD) were then recorded using a microplate spectrophotometer (GENios Tecan, Austria) at 590 nm. The results were expressed as a percentage of OD values of treated cell cultures compared to vehicle-treated ones.

### Real-time qPCR

After treatments, total RNA was isolated using the TRIZOL reagent (Life Technologies, Monza, Italy) according to the method of Chomczynski and Sacchi [41]. RNA integrity was checked by 1% agarose gel electrophoresis and RNA concentrations were measured by spectrophotometry (all RNA samples displayed an OD260/OD280 ratio > 1.8 and <2.0). Total RNA was reverse transcribed with the GeneAmp RNA PCR kit (Life Technologies,) using random hexamers (0.75 μg of total RNA in a final reaction volume of 20 μl). Relative abundance of each mRNA species was assessed by real-time RT-PCR using the Syber Green gene expression Master Mix (Life Technologies) in a Step One Real-Time PCR System (Life Technologies,). All samples were run in triplicate and all data were normalized to glyceraldehyde-3-phosphate dehydrogenase (GAPDH) as the endogenous reference gene. Relative expression of different gene transcripts was calculated by the Delta-Delta Ct (DDCt) method and converted to relative expression ratio (2^−DDCt^) for statistical analysis [42]. The primers used for PCR amplification were designed using Primer 3 [43] and their sequences have been previously validated (see Table 2) [28, 38].

**Table 2:** Primer sequences used for real-time qPCR

Gene	Forward (5′ – 3′)	Reverse (5′ – 3′)	Product size
MOP	ATCACGATCATGGCCCTCTACTCC	TGGTGGCAGTCTTCATCTTGGTGT	106
NOP	GGCCTCTGTTGTCGGTGTTC	GTAGCAGACAGAGATGACGAGCAC	175
GAPDH	GGTCGGAGTCAACGGATTT	TGGACTCCACGACGTACTCA	281

*MOP* μ opioid receptor, *NOP* nociceptin/orphanin FQ opioid peptide receptor, *GAPDH* glyceraldehyde-3-phosphate dehydrogenase

### Statistical analysis

Data from MTT assay were statistically analysed by two-way analysis of variance (ANOVA). F-values reaching significance (*p* < 0.05) were further analyzed by Bonferroni post-hoc test. Data from gene expression were analyzed by one-way ANOVA, followed by Newman-Keuls test. Statistical analysis was performed using the Graph-Pad Prism software v. 5 (GraphPad Software, San Diego, CA, USA). Results are reported as the mean of values ± SEM (n/assay = 4).

## Results

### Phytochemical analysis of WSE

**Fig. 1:**
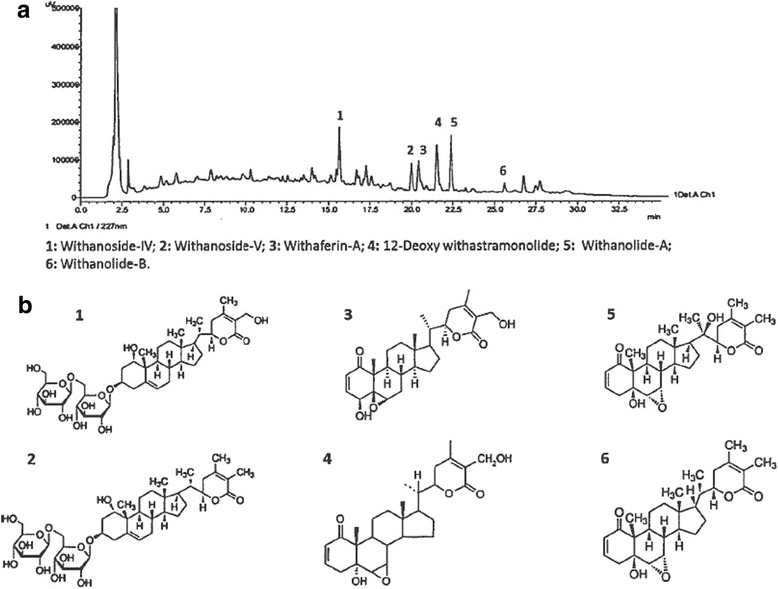
Chromatogram of WSE (**a**) obtained using an HPLC system (Shimadzu, LC 2010 A, Japan) equipped with UV detector, auto-injector, and column oven with class VP software (see Methods for details); numbers above peaks refer to the withanolides reported in the lower panel of the figure (**b**)

HPLC-fingerprint analysis of WSE indicated the presence of the following withanolides: withanoside-IV, withanoside-V, withaferin-A, 12-deoxy withastramonolide, withanolide-A, and withanolide-B (Fig.1a and 1b). Their individual concentrations, expressed as % *w*/w, are reported in the Table 3; the global content of identified withanolides in WSE was >2.5% w/w.

**Table 3:** Phytochemical analysis of *Withania somnifera* root extract by HPLC

Analyte	Concentration (% w/w)
a) Withanoside-IV	0.88
b) Withanoside-V	0.47
c) Withaferin-A	0.66
d) 12-Deoxy withastramonolide	0.33
e) Withanolide-A	0.41
f) Withanolide-B	0.07
Sum of withanolides conc.’s (w/w)	2.82

(Batch No.: WS/11003; Lab Reference/Report No.: FP101002)

### MTT assay for cell viability

SH-SY5Y cells exposed to WSE showed not significant alterations of cell survival following 5 h (Table 4). On the contrary, 24 and 48 h of exposure caused a dose-dependent cell viability reduction which appeared more pronounced overtime (Table 4). Two-way ANOVA revealed a significant time × treatment interaction [F_(10,51)_ = 8.23; *p* < 0.0001]. Since 24 and 48 h exposure to WSE significantly decreased SH-SY5Y viability; these time-points were excluded from the subsequent gene expression analyses that were, accordingly, conducted following 5 h exposure only.

**Table 4:** Effects of *Withania Somnifera* root extract on cell viability in the SH-SY5Y cells

	Time of exposure		
	5 h	24 h	48 h
Vehicle	100.00 ± 3.64	100.00 ± 12.62	100.00 ± 15.10
0.10 mg/ml WSE	91.83 ± 14.38	90.52 ± 15.53	79.65 ± 14.40
0.25 mg/ml WSE	93.90 ± 5.40	72.63 ± 4.16	14.11 ± 1.43***
0.50 mg/ml WSE	107.21 ± 6.41	49.35 ± 3.79***	15.02 ± 4.13***
0.75 mg/ml WSE	107.04 ± 5.35	51.73 ± 2.69***	7.41 ± 3.02***
1.00 mg/ml WSE	109.90 ± 6.23	34.69 ± 6.29***	7.62 ± 2.76***

Data are expressed as mean ± SEM (*** *p* < 0.001 vs vehicle are indicated in bold) and analyzed by two-way ANOVA. F-values reaching significance (*p* < 0.05) were further analysed by Bonferroni post-hoc test

### MOP and NOP gene expression analysis

#### Treatment a: Morphine and naloxone induce opposite effects on MOP and NOP gene expression

**Fig. 2:**
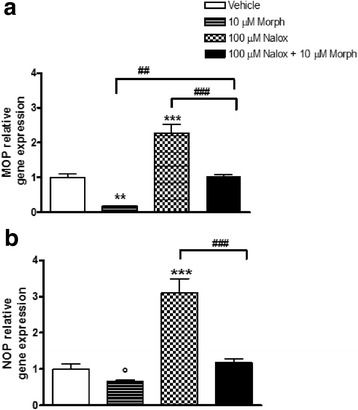
MOP (**a**) and NOP (**b**) relative gene expression in SH-SY5Y neuroblastoma cells following 5 h of exposure to 10 μM morphine, 100 μM naloxone and their association. Data represent 2^−DDCt^ values calculated by using the DDCt method. Gene expression was normalized to GAPDH as housekeeping gene. Data are expressed as mean ± SEM (° *p* = 0.074; ** *p* < 0.01, *** *p* < 0.001 vs vehicle; ## *p* < 0.01 and ### *p* < 0.001 vs naloxone + morphine; data analyzed by one-way ANOVA followed by Newman-Keuls tests)

A significant MOP gene expression down-regulation was observed following 5 h of 10 μM morphine exposure (0.17 ± 0.01 vs vehicle 1.00 ± 0.11, *p* < 0.01; Fig. 2a). Conversely, 100 μM naloxone induced a significant MOP mRNA increase (2.27 ± 0.25 vs vehicle 1.00 ± 0.11, *p* < 0.001; Fig. 2a). The co-exposure to morphine and naloxone did not change MOP gene expression compared to vehicle (Fig. 2a); however, the effects induced by morphine or naloxone alone were significantly different with respect to those observed after their co-exposure (*p* < 0.01, *p* < 0.001; Fig. 2a). A trend of reduction for NOP receptor gene expression was observed after morphine exposure (0.66 ± 0.03 vs vehicle 1.00 ± 0.14, *p* = 0.074; Fig. 2b), whereas naloxone induced a significant NOP up-regulation (3.10 ± 0.39 vs vehicle 1.00 ± 0.14, p < 0.01; Fig. 2b). NOP gene expression analysis after naloxone and morphine co-exposure did not show significant alterations compared to vehicle (Fig. 2b); however, statistically significant differences were observed between the effects induced by the co-exposure and those induced by the exposure to naloxone alone (*p* < 0.001; Fig. 2b).

#### Treatment B: WSE causes selective alterations of MOP and NOP gene expression

**Fig. 3:**
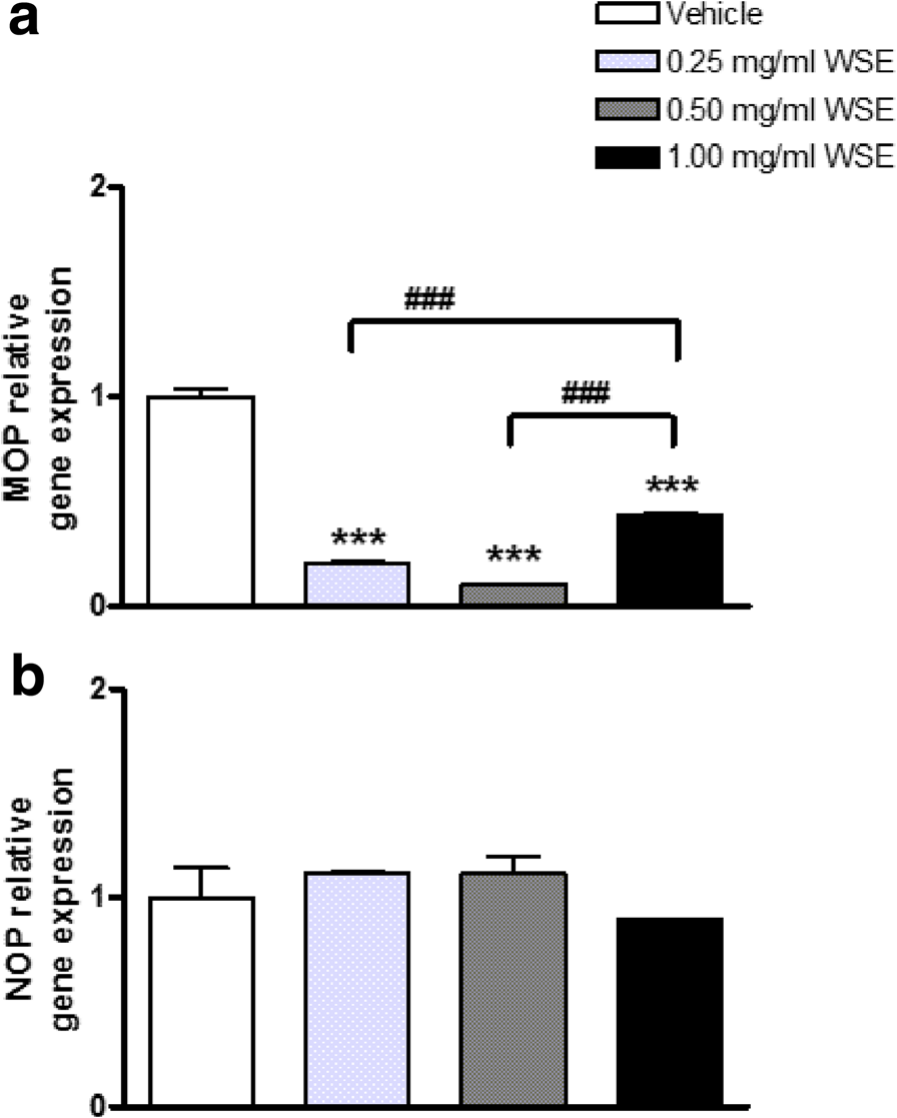
MOP (**a**) and NOP (**b**) relative gene expression in SH-SY5Y neuroblastoma cells following 5 h of exposure to WSE (0.25 mg/ml, 0.50 mg/ml and 1.00 mg/ml). Data represent 2^−DDCt^ values calculated by using the DDCt method. Gene expression was normalized to GAPDH as housekeeping gene. Data are expressed as mean ± SEM (***p < 0.001 vs vehicle, ### *p* < 0.001 vs 1.00 mg/ml WSE; data analyzed by one-way ANOVA followed by Newman-Keuls tests)

WSE significantly reduced MOP gene expression levels at all concentrations used (0.25 mg/ml WSE: 0.20 ± 0.01; 0.50 mg/ml WSE: 0.10 ± 0.01; 1.00 mg/ml WSE: 0.43 ± 0.01 vs vehicle: 1.00 ± 0.04, respectively, *p* < 0.001; Fig. 3a). In addition, the decrease induced by 1.00 mg/ml WSE was also significantly different from those induced by the doses of 0.25 and 0.50 mg/ml (*p* < 0.001). On the contrary, no changes of NOP mRNA levels were caused by WSE at any concentration (Fig. 3b).

#### Treatments C and D: WSE modifies morphine and naloxone effects on MOP and NOP gene expression

**Fig. 4:**
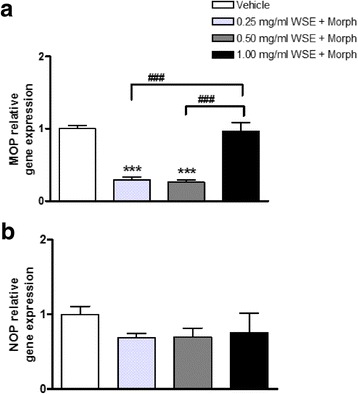
MOP (**a**) and NOP (**b**) relative gene expression in SH-SY5Y neuroblastoma cells following 5 h of exposure to WSE (0.25 mg/ml, 0.50 mg/ml and 1.00 mg/ml) in association to 10 μM morphine. Data represent 2^−DDCt^ values calculated by using the DDCt method. Gene expression was normalized to GAPDH as housekeeping gene. Data are expressed as mean ± SEM (***p < 0.001 vs vehicle; ###*p* < 0.001 vs 1.00 mg/ml WSE + morphine; data analyzed by one-way ANOVA followed by Newman-Keuls tests)

WSE at 0.25 and 0.50 mg/ml failed to alter the ability of 10 μM morphine to decrease MOP gene expression (0.25 mg/ml WSE + morphine: 0.30 ± 0.04; 0.50 mg/ml WSE + morphine: 0.26 ± 0.03 vs vehicle 1.00 ± 0.05, respectively; *p* < 0.001; Fig. 4a); on the contrary, the highest WSE concentration (1.00 mg/ml) significantly obstructed the morphine-induced MOP down-regulation (Fig. 4a).

Moreover, the effects induced by 1.00 mg/ml WSE + morphine resulted significantly different from those induced by WSE at 0.25 or 0.50 mg/ml + morphine (*p* < 0.001; Fig. 4a). In contrast, WSE pre-treatment 30 min before morphine addition to cell cultures failed to significantly affect NOP receptor gene expression changes (Fig. 4b).

**Fig. 5:**
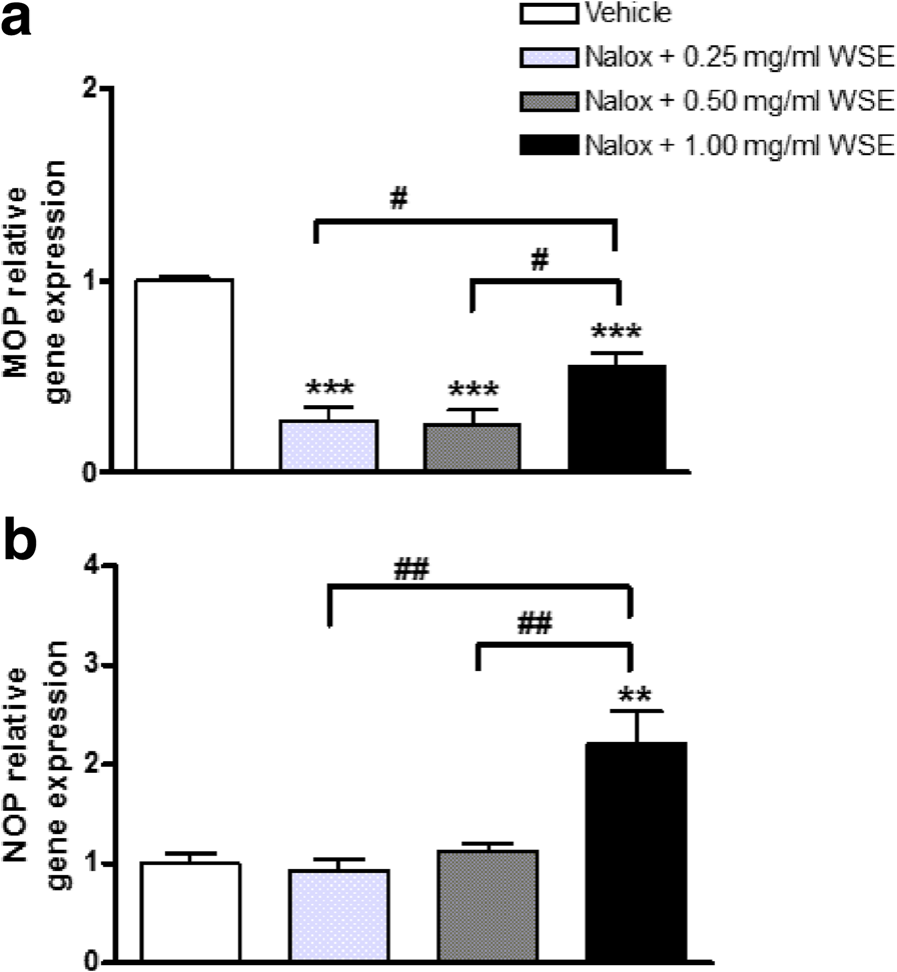
MOP (**a**) and NOP (**b**) relative gene expression in SH-SY5Y neuroblastoma cells after 5 h of 100 μM naloxone and WSE exposure (0.25 mg/ml, 0.50 mg/ml and 1.00 mg/ml). Data represent 2^−DDCt^ values calculated by using the DDCt method. Gene expression was normalized to GAPDH as housekeeping gene. Data are expressed as mean ± SEM (**p < 0.01, ***p < 0.001 vs vehicle, #*p* < 0.05, ##*p* < 0.01 vs naloxone +1.00 mg/ml WSE; data analyzed by one-way ANOVA followed by Newman-Keuls tests)

Interestingly, all WSE concentrations tested hampered the naloxone-induced MOP up-regulation, and indeed a significant decrease of MOP mRNA levels was observed resembling the effect caused by WSE alone (0.25 mg/ml WSE + naloxone: 0.27 ± 0.07; 0.50 mg/ml WSE + naloxone: 0.25 ± 0.08; 1.00 mg/ml WSE + naloxone: 0.55 ± 0.07 vs vehicle 1.00 ± 0.02, respectively; *p* < 0.001; Fig. 5a). Moreover, significant differences were observed between the effects induced by naloxone +1.00 mg/ml WSE and those induced by naloxone +0.25 or 0.50 mg/ml WSE (*p* < 0.05 for both WSE concentrations; Fig. 5a).

Finally, NOP receptor gene expression analysis disclosed the ability of WSE, at its lower concentrations, to prevent the naloxone-induced NOP up-regulation; on the contrary, in the presence of the highest WSE concentration, naloxone was still able to significantly up-regulate NOP gene expression (2.20 ± 0.32 vs vehicle 1.00 ± 0.10; *p* < 0.01, Fig. 5b). Statistically significant differences were observed between the effects induced by naloxone +1.00 mg/ml WSE and those induced by naloxone +0.25 or 0.50 mg/ml WSE (*p* < 0.01 for both concentrations; Fig. 5b).

## Discussion

The modulation of morphine analgesic effect exhibited in-vivo by *Withania somnifera* [24, 25] could represent an useful tool to improve the opioid-sparing strategies in pain therapy. However, little is known about how WSE can modify the molecular mechanisms leading to the development of tolerance, which represents one of the major limitations in opiate clinical use.

On these bases, since cellular adaptations responsible for tolerance development can include gene expression alterations [26], the present study investigated the effects induced by WSE on the MOP and NOP receptor mRNA levels in neuroblastoma SH-SY5Y cells.

To this end, a range of WSE concentrations was tested by MTT assay showing that the concentrations of WSE examined were not cytotoxic up to 5 h of exposure. In contrast, the increase of the exposure time revealed a significant decrease of cell viability. Based on MTT results, 5 h was the interval selected to perform the gene expression analyses since at this time a general toxicity response could be ruled out in the interpretation of the findings.

The first experiment (treatment A, see Methods) showed that morphine elicited MOP gene expression down regulation and NOP receptor reduction, though not significant, whereas naloxone evoked a significant increase of their mRNA levels. The finding of morphine-induced MOP down-regulation is in agreement with previous data obtained from our [27] and other laboratories [44, 45] in SH-SY5Y, as well as in other cell lines [46, 47].

Our data additionally demonstrated that, in agreement with previous studies [46], morphine-induced MOP down-regulation was inhibited by naloxone pre-treatment. However, the effect of naloxone on opioid receptor gene expression were not restricted to the receptor antagonism activity; in fact, naloxone alone significantly up-regulated MOP and NOP gene expression. This peculiar ability of naloxone has been observed in some previous studies. In particular, Gach et al. [46] reported that naloxone alone produced an approximately 20% increase of MOP mRNA levels as well as a 68% increase in MOP protein levels in MCF-7 cells. Moreover, the prolonged intracerebroventricular infusion of naloxone or naltrexone has been reported to cause a marked up-regulation of prodynorphin gene expression in selected rat brain areas [48].

The results of second part of the study (treatment B, see Methods), in which we tested the effects of WSE (0.25, 0.50 and 1.00 mg/ml)on the MOP and NOP gene expression indicate for the first time that WSE induced selective alterations of opioid receptor mRNA levels. In particular, we observed a significant decrease of MOP gene expression without alterations of NOP mRNA levels. Notably, the WSE-induced MOP down-regulation appeared significantly more pronounced at the lower concentrations than at the highest one. Several issues arise from these results. First, WSE clearly reduces, in a dose-independent manner, MOP mRNA levels only. It is conceivable that the different effects caused by diverse WSE concentrations might depend on the presence of multiple components endowed with diverse activities. In this frame, the lack of a dose-dependent effect suggests the existence of a complex interaction among the different components in the extract. In fact, WSE is a root extract containing many chemical constituents, of which 12 alkaloids and 35 withanolides [49, 50], responsible for its multiple medicinal properties. In this regard, it is worth considering that there are instances in which the use of isolated single constituents of an herbal preparation does not precisely reproduce the therapeutic profile of the whole extract. For example, although the antidepressant properties ascribed to the use of *Hypericum perforatum* (Saint John’s Wort) are recognized to be mainly due to hypericin and hyperforin [51], the literature suggests caution in interpreting the results obtained following the administration of different extracts [52] or individual compounds [53], because of their peculiar pharmacokinetic and pharmacodynamic properties [52, 54].

The MOP gene expression observed after exposure to WSE could help understanding the data previously reported by Kulkarni and Ninan [24]. These authors observed the lack of morphine analgesic effect when the opiate was administered to mice that had previously received 100 mg/kg WSE for nine days. Based on our results, it is conceivable that morphine inefficacy could depend on MOP down-regulation induced by WSE.

A peculiar alteration of MOP gene expression emerged after treatment C. In these experiments cells exposed to morphine following a WSE pre-treatment for a total period of 5 h still exhibited a significant MOP mRNA decrease after lower WSE concentrations. In contrast, at the highest WSE concentration we observed a lack of MOP receptor down-regulation, an observation that highlights the peculiar feature of this (1.00 mg/ml) WSE concentration. This result could explain how the tolerance to morphine analgesic effect was hampered when WSE (100 mg/kg i.p.) was injected 30 min before morphine [24]. Therefore, based on these two observations, it can be hypothesized that the simultaneous presence of high WSE concentration and morphine could somehow be responsible of maintaining an adequate MOP receptor amount sufficient to produce the analgesic response.

The absence of NOP alterations induced by WSE alone represents an additional point of interest. In fact, previous studies suggested that MOP and NOP receptors are both involved in tolerance development. Notably, NOP receptor knockout mice display a partial loss of morphine tolerance [55]. In this frame, recent results obtained in our laboratory showed that fentanyl as well as the 14-O-Methylmorphine-6-sulfate, two potent analgesic agents endowed with lower tolerance to the analgesic effect than morphine, did not modify NOP receptor gene expression [27, 38]. Hence, since WSE pre-treatment followed by morphine exposure disclosed the potential ability of WSE to hinder the morphine-induced NOP alteration, these results could be related to a less rapid onset of morphine tolerance in the presence of WSE. In fact, the recruitment of the nociceptin/orphanin FQ (N/OFQ) - NOP system could be functional to the occurrence of morphine tolerance, and the N/OFQ antagonists may prevent tolerance development [56–58].

Finally, since we observed that opioid receptor gene expression can be increased by the competitive antagonist naloxone, we also evaluated whether WSE may influence these naloxone’s effects. Results showed that WSE hampered naloxone-elicited MOP up-regulation and prevented NOP up-regulation. However, this last effect on NOP was elicited by only the lower WSE concentrations but not by the highest one thus underlining, once again, the different effect produced by WSE at its highest concentration (1.00 mg/ml).

Interestingly, G protein-coupled receptors (GPCRs), such as MOP and NOP, can adopt multiple active conformations that combine to a diverse set of downstream effectors and structurally distinct ligands can preferentially activate a subset of intracellular signaling cascades (so called biased ligands) [59]. In this regard, since WSE displays a moderate affinity for opioid receptors, it is conceivable that its interaction could interfere with the intracellular signaling triggered by the opioid ligands. Moreover, also the posttranslational control of GPCRs seems to be crucial; in fact, the gene expression regulation achievable by targeting mRNAs could be a promising candidate to coordinate the complex response to analgesic drugs [60, 61]. Additional research will be necessary to fully elucidate the opioid receptor transcriptional regulation and the downstream signaling influenced by the WSE/morphine interplay.

## Conclusions

In summary, present in-vitro data suggest that the ability of WSE to affect morphine analgesic profile could take place through the gene expression regulation. In this regard, it has been hypothesized that the altered gene expression is a likely process for inducing neuroadaptations responsible for tolerance. In particular, the reduction of dendritic spine density has been correlated with the development of morphine tolerance and this neuroadaptation is regulated at gene expression level [26]. Since WSE ability to counteract morphine-induced spine density reduction upon withdrawal [22], as well as its morphine tolerance counteracting action have been demonstrated [24], it is conceivable that the mechanism by which WSE exerts these effects can include the opioid receptor gene expression regulation. In conclusion, our results showed that WSE may influence the opioid receptor gene expression and they offer new information about the complex interaction of the WSE with opioid ligands’ effects on the MOP and NOP biosynthesis.

## Abbreviations

DDCt: Delta–Delta threshold cycle
FBS: Fetal bovine serum
GAPDH: Glyceraldehyde-3-phosphate dehydrogenase
MOP: μ opioid receptor
MTT: [3-(4,5-dimethylthiazol-2-yl)-2,5-diphenyltetrazolium bromide]
N/OFQ: Nociception/orphanin FQ
NOP: Nociceptin/orphanin FQ opioid peptide receptor
OD: Optical densities
WSE: *Withania somnifera* root extract
